# Non-fatal senior pickleball and tennis-related injuries treated in United States emergency departments, 2010–2019

**DOI:** 10.1186/s40621-021-00327-9

**Published:** 2021-05-03

**Authors:** Harold Weiss, Jacob Dougherty, Charles DiMaggio

**Affiliations:** 1 Department of Population Health Sciences, University of Wisconsin, Madison, WI USA; 2 Chicago, USA; 3 Department of Surgery, NYU Grossman School of Medicine, New York, USA

**Keywords:** Pickleball, Tennis, Sports and recreation, Physical activity, Falls, Fractures, Seniors, Injury, Emergency department, NEISS

## Abstract

**Background:**

Pickleball is growing rapidly with a passionate senior following. Understanding and comparing players’ injury experience through analysis of a nationally representative hospital emergency department sample helps inform senior injury prevention and fitness goals.

**Methods:**

A cross-sectional descriptive study was performed using 2010 to 2019 data from the U.S. Consumer Product Safety Commission’s (CPSC) National Electronic Injury Surveillance System (NEISS). Tennis was selected for comparison purposes because of the similarity of play, occasional competition for the same court space, and because many seniors play both sports. Non-fatal pickleball and tennis-related cases were identified, examined, recoded, and separated by injury versus non-injury conditions. Since over 85% of the pickleball injury-related cases were to players ≥60 years of age, we mostly focused on this older age group. Analyses consisted of descriptive statistics, injury frequency, type and trends over time, and comparative measures of risk.

**Results:**

Among players ≥60 years of age, non-injuries (i.e., cardiovascular events) accounted for 11.1 and 21.5% of the pickleball and tennis-related cases, respectively. With non-injuries removed for seniors (≥60 years), the NEISS contained a weighted total of 28,984 pickleball injuries (95% confidence interval [CI] = 19,463–43,163) and 58,836 tennis injuries (95% CI = 44,861-77,164). Pickleball-related injuries grew rapidly over the study period, and by 2018 the annual number of senior pickleball injuries reached parity with senior tennis-related injuries. Pickleball-related Slip/Trip/Fall/Dive injury mechanisms predominated (63.3, 95% CI = 57.7–69.5%). The leading pickleball-related diagnoses were strains/sprains (33.2, 95% CI = 27.8–39.5%), fractures (28.1, 95% CI = 24.3–32.4%) and contusions (10.6, 95% CI = 8.0–14.1%). Senior males were three-and-a-half times more likely than females to suffer a pickleball-related strain or sprain (Odds Ratio [OR] 3.5, 95% CI = 2.2–5.6) whereas women were over three-and-a-half times more likely to suffer a fracture (OR 3.7, 95% CI = 2.3–5.7) compared to men and nine times more likely to suffer a wrist fracture (OR 9.3 95% CI = 3.6–23.9). Patterns of senior tennis and pickleball injuries were mostly similar.

**Conclusions:**

NEISS is a valuable data source for describing the epidemiology of recreational injuries. However, careful case definitions are necessary when examining records involving older populations as non-injury conditions related to the activity/product codes of interest are frequent. As pickleball gains in popularity among active seniors, it is becoming an increasingly important cause of injury. Identifying and describing the most common types of injuries may can help inform prevention and safety measures.

## Background

### Pickleball background

Pickleball is emerging as a timely and important injury topic due to its ease of play, exercise benefits, rapid growth and passionate following among seniors (Forrester 2020). These factors raise important questions for researchers, clinicians and participants related to injury vulnerability and risks, injury prevention, cardiovascular risks, senior fitness and participant well-being.

Pickleball was developed in the United States (U.S.) in 1965 (Greiner 2019; USA Pickleball Association (USAPA) 2020). It is played either indoors or outdoors on a badminton-sized court with a net slightly lower than a tennis net. A non-volley zone (“the kitchen”) extends seven feet from the net on each side; its effect being to slow down play, encourage softer paddle strokes and reduce high-speed ball returns (“smashes”) (Vitale and Liu 2020). Pickleball is played on repurposed tennis courts or increasingly on dedicated court facilities. Pickleball equipment is simple with a lightweight paddle usually comprised of composite materials with a cost ranging from $50–$150+ and a light plastic baseball-sized whiffle-ball (0.78 to 0.935 oz) (USA Pickleball Association (USAPA) 2020). It is usually played as a doubles game (two players to a team, mixed or gender specific), but can also be played as a singles or triples contest. Individual games last around 10 to 20 min depending on the match and scoring system in use. Most core players play several games during a one to two-hour or more session with short rests between games. It is played mainly as a social sport, attracting participants of all ages, fitness levels and abilities while also played as a family and school recreational activity. Professional competitions are increasingly popular with tournaments across the U.S. and Canada (PickleballTournaments.com 2020).

The Sports & Fitness Industry Association (SFIA) reported pickleball had 3.3 million players in the U.S. in 2019 (Sports and Fitness Industry Association’s (SFIA) 2019). Of these, 2.0 million were classified as “casual” participants (playing one to seven times a year) and 1.3 million were “core” participants (playing eight or more times a year). The average annual growth rate for all age players from 2015 to 2018 was 9.7% with the highest average annual growth rate of 39.6% among core players ≥65 years of age.

Comparisons to tennis are warranted because of the similarity of play, occasional play and competition for the same indoor and outdoor court space, and because many seniors play both sports (Buzzelli and Draper 2020; Casper and Jeon 2017; Heo et al. 2018a). The Tennis Industry Association (TIA) reported that tennis had 17.84 million players in 2019 with a mostly flat participation rate over the past few years (Tennis Industry Association 2019). Of these, 1.88 million (10.5%) were 55 years or older (Tennis Industry Association 2019). In contrast, pickleball is rapidly growing and extremely popular among seniors. In 2019, the TIA reported there were 1.15 million pickleball players 55 years or older out of 3.3 million (35.1%) (USA Pickleball Association (USAPA) 2020).

### Importance of research

There are few analyses or published papers addressing pickleball injuries. Most of the literature has focused on psychosocial, well-being and fitness aspects (Buzzelli and Draper 2020; Casper and Jeon 2017; Heo et al. 2018a; Heo et al. 2018b; Ryu et al. 2020). Other than a few case reports, (Vitale and Liu 2020; Atkinson et al. 2020) there have been only three papers addressing injury risk: Greiner (2019), Quail (2019) and Forrester (2020) (Forrester 2020; Greiner 2019; Quail 2019). Greiner published a short review that discussed injury aspects relative to other racquet sports (Greiner 2019). It speculated on acute and chronic injury-related movement, but did not cite any case data or include discussion of equipment or risk factors. Quail’s paper focused on clinical aspects of the topic (Quail 2019) but like Greiner did not describe specific injuries from playing pickleball. Forrester examined U.S. Consumer Product Safety Commission’s (CPSC) National Electronic Injury Surveillance System (NEISS) data query system pickleball-related data from 2001 to 2017 (Forrester 2020). He also compared pickleball findings to the literature on other racquet sports. Our study updates and refines Forrester’s paper with more stringent case definitions, use of sample derived point estimates and confidence intervals, assignment of the mechanism of injury and age-specific comparisons to tennis.

## Methods

This was a cross-sectional descriptive study using publicly available online data from CPSC’s NEISS emergency department data query system (U.S. Consumer Product Safety Commission 2020a). This data set contains a nationally representative validated probability sample from about 100 of the > 5000 U.S hospitals providing emergency services. The NEISS sample is stratified based on hospital size and age served (non-specialized versus children’s hospitals). Detailed NEISS data collection and sampling procedures, changes over time, and statistical handling aspects have been reported elsewhere (U.S. Consumer Product Safety Commission 2020a; U.S. Consumer Product Safety Commission 2020b; Schroeder and Ault 2001).

### Data source and variables

Cases from the ten-year period 2010 to 2019 (unweighted *n* = 3,782,633) were downloaded from NEISS in comma separated value (CSV) format and imported into statistical packages for query, review, filtering, formatting, recoding, sub-setting, and analysis. Variables examined included treatment date, age, gender, body-part affected, primary diagnosis, disposition, location type and the involved product/activity (product codes). Each NEISS record also contains a 400-character narrative text field containing descriptive comments derived from the emergency department (ED) record about the patient, involved product(s)/activity, sequence of events, associated diagnoses, affected body part(s), and other information (U.S. Consumer Product Safety Commission 2019). The narrative text field was used to find pickleball cases since there is no specific product/activity code for pickleball, refine case selection, and to assign the mechanism of injury.

### Case selection

**Fig. 1:**
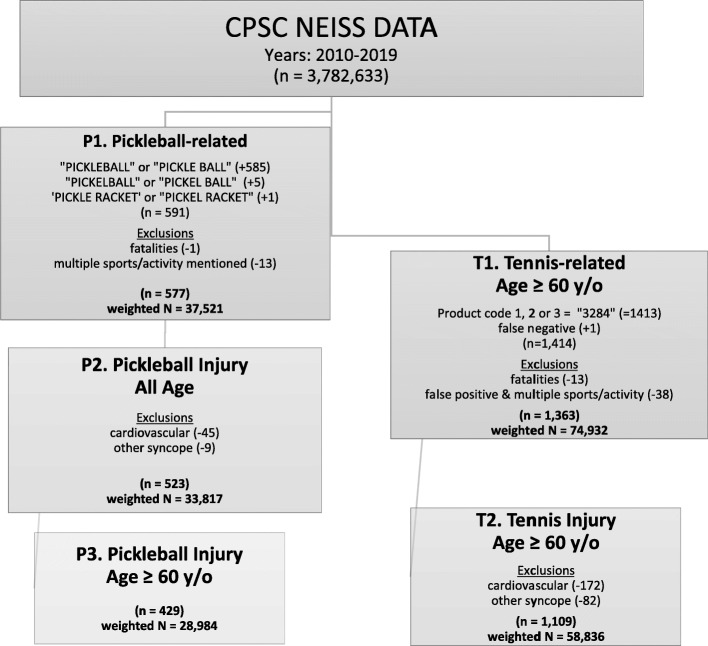
Case and group selection strategy and results. Numbers in parentheses refers to unweighted case counts (lower case n) while upper case N refers to weighted counts. “y/o” = year old

Because NEISS does not capture fatalities well, (Acton et al. 2019) and deaths in this sport-specific study were few and rarely if ever injury related, fatalities were excluded from this study. Case selection procedures are summarized in Fig. 1.

#### Pickleball

Pickleball does not have a specific NEISS product/activity code. Most pickleball-related cases were assigned a non-specific product code of 3235 (“Other ball sports, activity/apparel/equipment”). Potential cases were selected with a computer assisted search of the narrative text field for the following case-insensitive strings: “PICKLEBALL” or “PICKLE BALL” (*n* = 585), the common misspellings “PICKELBALL” or “PICKEL BALL” (n = 5) and “PICKLE RACKET”’ or “PICKLE RACQUET” (*n* = 1).

Records were excluded if: a) fatal (unweighted n = 1), b) if more than one sport or activity was mentioned in the narrative related to the injury (unweighted *n* = 13) and c) a special case containing the word “PICKLE” (described in the limitations section). These criteria resulted in the initial selection of group P1 from the 2010–2019 NEISS data of *n* = 577 unweighted records (weighted *N* = 37,521, 95% CI = 25,005–56,301).

An important case definition issue with NEISS data, especially pertinent to an older cohort, is that NEISS may contain non-injury events if patients were engaged in the sport/activity at the time an acute non-injury medical condition appeared. For example, chest pain can be caused by either an injury or a cardiovascular condition acquired while playing pickleball. From a sports medicine perspective, emergency non-injury events related to a sport or activity are of interest. Given the predominance of older persons among pickleball participants, a significant number of diagnoses in both pickleball and older tennis age subsets included many non-injury medical conditions such as syncope (fainting), chest pain unrelated to trauma and cardiovascular events and related symptoms (such as atrial fibrillation, cardiac arrest, tachycardia, etc.).

These case-definition considerations were addressed by enumerating and briefly describing the cases with non-injury-related syncope (unweighted *n* = 9) and cardiovascular mentions (without indication of traumatic injury) in the record narrative (unweighted *n* = 45) but excluding them from the injury analyses. Note that mentions of dehydration and heat exhaustion were included as injuries since “heat-related illnesses” are considered injuries in traditional injury epidemiology classifications. Therefore, if the symptom of “syncope” was stated in the narrative as related to a body temperature elevation or dehydration, they were included and assigned to the heat-related illness mechanism. However, other cases with syncope or dizziness reported *without mention* of heat, dehydration, injury, or cardiovascular issues were excluded from the injury subsets. After these exclusions, the all-age pickleball injury cohort (group P2) consisted of 523 unweighted records (weighted *N* = 33,817, 95% CI = 22,942–49,847).

Lastly, pickleball injuries involving cases ≥60 years of age were selected for the primary analyses and comparisons. The age criteria was based on the knowledge that the average retirement age in the U. S, among living retirees in 2019 was 59.9 years (PK 2019) and the convenience of working with 10-year age groups for rate calculations. This left an unweighted *n* = 429 senior pickleball injury-related records (weighted *N* = 28,984, 95% CI = 19,463–43,163) in group P3.

#### Senior tennis comparison group

For comparison purposes, NEISS tennis injuries ≥60 years of age were selected over the same period by searching for the code “3284” (tennis activity/apparel/equipment) in the three fields indicating the products involved. This resulted in an unweighted *n* = 1413 + 1 false negative found by looking at all mentions of the word “TENNIS” in the narrative text field. However, the “3284” code incorporates not just the “activity” of “tennis” but also any tennis-related apparel or equipment involved in an injury. Therefore, we distinguished “playing tennis” as a recreational activity, from injuries involving tennis-related equipment while not engaged in playing tennis. Manual review of each senior tennis-related narrative for cases with a product code of “3284” led to many such exclusions. For example, a child struck in the eye at home with a “tennis ball”, lacerations involving “cutting tennis balls”, or falls related to “tennis balls” on an elderly assistive “walker” were all excluded.

Like the pickleball case selection, for the senior tennis-related cases we excluded fatalities (unweighted *n* = 13), one false positive and the multiple sport mentions (unweighted *n* = 35). This left an unweighted *n* = 1363 senior tennis related cases (weighted *N* = 74,932, 95% CI = 55,580-101,021) in group T1. Lastly, tennis cases with non-injury syncope (unweighted *n* = 82) and cardiovascular mentions without indication of traumatic injury in the record narrative (unweighted *n* = 172) were removed. This left *n* = 1109 records (weighted *N* = 58,836, 95% CI 44,736-77,381) in the senior tennis-related injury cohort, group T2.

### Mechanism of injury and variable regrouping

Certain variables were created or regrouped from the NEISS data as follows:

#### Primary injury mechanism assignment

Each tennis and pickleball-related case narrative text was used to assign an injury mechanism based on a modification of the coding scheme used by Gaw et al. (Gaw et al. 2014) to describe how the injury took place. In instances of narrative overlap, the first mechanism described was coded that most directly related to the injury. The case narrative text review resulted in assigning each case to a primary mechanism (or to an exclusion category) as follows:

1. Slip/Trip/Fall/Dive. A combined category.
2. Hit with racket or paddle (or “bat” if pickleball was mentioned).
3. Hit with ball.
4. Other specified mechanism. Commonly included a movement of some type (i.e., sudden stop, lunging, running, bending over, hyperextending, dislocation, sprain, twist, strain, bump, tear, pull, sudden pop or snap, inverted or rolled ankle), and less common mechanisms such as jammed body part, cutting a finger, abrasion, or insect sting.
5. Play/playing tennis or pickleball. Encompassed injuries that incurred during the activity where the mechanism could not be determined or was unknown (i.e., musculoskeletal pain, contusion, or epistaxis).
6. Heat-related illness. Assigned only if syncope/dizziness *and* hot/heat or dehydration was mentioned.
7. Hit with, by, tripped or ran into other object or slipped on other equipment. Includes hitting fence, net, wall, chair, bench, tree, tripped or fell over ball, racquet, or paddle.
8. Hit with, by or ran into another player.

Exclusions were assigned as follows:

- The case involved multiple sports mentions or was not a tennis or pickleball “playing” activity.
- Other syncope, dizziness, or dyspnea *without* mention of heat, injury, or cardiovascular issue.
- Possible cardiovascular event. This includes heart rhythm issue, angina, chest pain, deep vein thrombosis (dvt), blood in urine, altered mental status (AMS), pleural effusion, gastro-intestinal (GI) bleed, ataxia, weakness, or abnormal blood pressure *without* mention of traumatic injury.

Special cases:

- A subarachnoid hemorrhage can be due to a ruptured aneurysm, an arteriovenous malformation (AVM), or a traumatic head injury. One such tennis-related case was included as a Slip/Trip/Fall/Dive since the narrative said they fell while playing tennis, but it is acknowledged that it was not well differentiated whether the hemorrhage preceded or followed the fall.
- It was assumed if the tennis-related case was coded as “3284” and the narrative said the person had been hit by a tennis racquet that they were playing tennis if there was no place of injury mentioned.

The primary mechanisms for all pickleball and senior tennis-related records were assigned independently by authors HW and JD. For pickleball cases, initial (unweighted) inter-rater reliability for the mechanism recoding was 94.9%. The cases in which the primary mechanism was assigned differently (discordant pairs) were all resolved by joint discussion. For tennis cases, inter-rater reliability for the mechanism recoding for the first independent review was 94.3% with all discordant pairs jointly resolved.

#### Body region

Body region injured was regrouped into: (1) upper extremity (including the NEISS categories of shoulder, elbow, upper arm, lower arm, wrist, hand, and finger); (2) lower extremity (including knee, upper leg, lower leg, ankle, foot, and toe); (3) trunk (including upper trunk, lower trunk, and pubic region); (4) head/neck (including head, face, eye, mouth, neck, and ear); and (5) other (including internal organs and injury to greater than 25% of the body) (Gaw et al. 2014).

An eye injury flag was assigned based on both the body part code (77 - EYEBALL) plus a search in the narrative text field for eye injury since sometimes the body part was assigned to the face or another body part code while the narrative text indicated an eye injury had occurred. Each narrative was reviewed, and the case included as an eye injury if it appeared the person has been struck in the eye resulting in injury (senior pickleball *n* = 4, senior tennis *n* = 20).

#### Disposition from the ED

Disposition from the ED was regrouped into 3 categories: (1) released; (2) hospitalized (including NEISS variables of treated and transferred, treated, and admitted, and held for < 24 h for observation); and (3) left against medical advice.

#### Location of injury

Location of injury was regrouped into school/public property, sports/recreation place, and other (including the NEISS categories of home, farm, apartment/condo, and street/highway).

### Analysis

Descriptive analyses consisted of unweighted record counts (n) and weighted counts (N) where indicated. Weighted stratified survey specific analyses, confidence interval calculations and table and graphics preparation were performed using the R statistical programming language and the ‘survey’, ‘lubridate’, ‘vroom’, ‘segmented’, and ‘ggpubr’ add-on packages (R Core Team 2013).

The methods used to identify statistically significant trends in senior pickleball injuries were drawn from Thomas Yokota’s example reproducing the Centers for Disease Controls’ (CDC) guide on conducting statistical trend tests with multiple years of complex survey data (Yokota n.d.). The R ‘segmented’ package was used to estimate breakpoints in the trend analysis, and data manipulation was performed using packages in the ‘tidyverse’ ecosystem (Wickham et al. 2019). Confidence intervals for case counts were calculated on a log scale which produces intervals close to the Coefficient of Variation referenced in the NEISS research guide (U.S. Consumer Product Safety Commission Division of Hazard and Injury Data Systems 2000; Lumley 2020).

Simple logistic regression was used to compute the Odds Ratios (ORs) to estimate the strength of associations between binomial outcome variables for senior pickleball versus senior tennis-related injuries. They were calculated using survey adjusted general linear models with a logit link as per the methods of DiMaggio et al. (DiMaggio et al. 2019). Trends were visualized by plotting annual rates of injury counts per yearly census population estimates by age group. Population estimates by age group were obtained using the cencusapi R package (Recht 2020).

## Results

### Group P2: all age pickleball injuries

Removing non-injuries resulted in *n* = 523 unweighted cases (weighted *N* = 33,817, 95% CI = 22,942–49,847). Table 1 summarizes the main univariate findings among all age pickleball injury-related cases.

**Table 1:** Univariate findings among all age pickleball injuries

Variable Value	Unweighted Count	Weighted Count	Weighted Variable Proportion	Lower 95% CI Limit of Weighted Count	Upper 95% CI Limit of Weighted Count
**Age Group (years)**					
0–39	29	1249	3.7%	775	2012
40–49	17	805	2.4%	445	1459
50–59	48	2779	8.2%	1630	4737
60–69	234	15,738	46.5%	10,317	24,007
70–79	171	11,804	34.9%	7802	17,859
80+	24	1442	4.3%	748	2780
**Sex**					
Male	265	17,500	51.7%	11,677	26,226
Female	258	16,318	48.3%	11,092	24,006
**Treatment Year**					
2010	8	462	1.4%	299	715
2011	7	389	1.2%	136	1110
2012	4	171	0.5%	33	872
2013	12	611	1.8%	107	3476
2014	22	1227	3.6%	731	2060
2015	54	3764	11.1%	1698	8340
2016	67	4888	14.5%	2560	9333
2017	85	5578	16.5%	2311	13,462
2018	116	7314	21.6%	3043	17,577
2019	148	9414	27.8%	3513	25,226
**Treatment Month**					
Jan	64	4292	12.7%	2766	6660
Feb	68	4619	13.7%	2671	7989
Mar	75	5517	16.3%	3267	9319
Apr	51	3380	10.0%	2036	5610
May	37	2190	6.5%	1493	3213
Jun	23	1389	4.1%	792	2436
Jul	27	1336	4.0%	730	2444
Aug	22	1289	3.8%	789	2106
Sep	22	1232	3.6%	711	2135
Oct	33	1913	5.7%	1028	3558
Nov	46	3178	9.4%	1909	5291
Dec	55	3482	10.3%	2218	5467
**Disposition Group**					
Released	481	31,430	92.9%	21,454	46,045
Hospitalized	42	2387	7.1%	1333	4277
**Primary Mechanism**					
Slip/Trip/Fall/Dive	318	21,415	63.3%	13,892	33,012
Other mechanism	119	7647	22.6%	4879	11,985
Undetermined/unknown	39	1968	5.8%	1393	2781
Hit object	18	1138	3.4%	680	1903
Heat-related illness	9	750	2.2%	324	1735
Hit with racquet/paddle	10	513	1.5%	250	1054
Hit player	4	253	0.7%	87	740
Hit with ball	6	133	0.4%	47	377
**Diagnosis**					
Strain, sprain	170	11,212	33.2%	6897	18,228
Fracture	150	9497	28.1%	6313	14,289
Contusions, abrasions	55	3589	10.6%	2285	5638
Internal injury	51	3220	9.5%	1890	5486
Other	47	3007	8.9%	1951	4635
Laceration	27	1736	5.1%	1110	2715
Dislocation	15	1016	3.0%	584	1769
Concussion	3	270	0.8%	87	843
Hematoma	5	268	0.8%	98	732
**Body Region**					
Upper extremity	172	11,305	33.4%	7491	17,062
Lower extremity	156	9919	29.3%	6344	15,508
Head/neck	103	6369	18.8%	4420	9178
Trunk	82	5455	16.1%	3510	8477
All of body	10	769	2.3%	339	1744
**Body Part**					
Wrist	67	4458	13.2%	2556	7774
Lower leg	65	4368	12.9%	2522	7566
Head	63	4038	11.9%	2607	6255
Lower trunk	57	3923	11.6%	2521	6103
Ankle	35	2052	6.1%	1247	3377
Knee	33	1989	5.9%	1143	3460
Shoulder	27	1814	5.4%	1048	3141
Upper trunk	24	1517	4.5%	863	2667
Finger	21	1399	4.1%	912	2145
Face	21	1194	3.5%	774	1843
Upper arm	17	1056	3.1%	499	2231
Elbow	14	990	2.9%	548	1790
Lower arm	16	957	2.8%	504	1818
Foot	14	934	2.8%	453	1927
All of body	10	769	2.3%	339	1744
Hand	10	632	1.9%	325	1230
Neck	10	626	1.9%	332	1178
Upper leg	8	495	1.5%	246	997
Mouth	3	264	0.8%	85	819
Eyeball	5	232	0.7%	60	888
Toe	1	81	0.2%	11	574
Ear	1	16	0.0%	2	114
Pubic region	1	16	0.0%	2	111
**Weekend**					
Weekday injury	391	25,283	74.8%	17,080	37,424
Weekend injury	132	8534	25.2%	5694	12,792
**Eye Injury**					
Non-eye injury	517	33,569	99.3%	22,749	49,536
Eye injury	6	248	0.7%	70	875
**Location Group**					
School, sports, and public	431	28,505	84.3%	17,895	45,406
Other	92	5312	15.7%	3204	8806

**Fig. 2:**
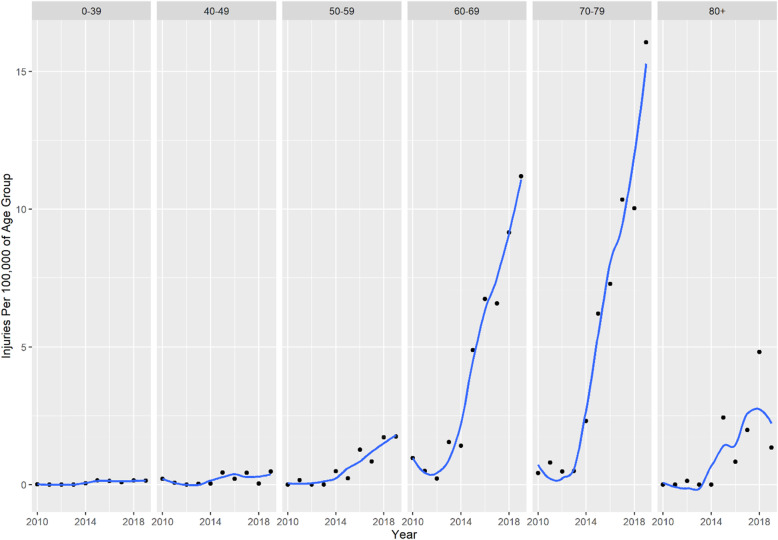
All Age Pickleball Injuries Per 100,000 of Age Group (weighted)

The average age for group P2 was 66 years (median age 68 years), and the 25th percentile was 63 years. Ages 60–79 made up 81.4% (95% CI = 76.8–86.1%) of all group P2 cases, growing rapidly since 2014. Figure 2 shows the rate by year of pickleball injuries for each age group.

The most common mechanism of injury was “Slip/Trip/Fall/Dive” (63.3% of weighted cases, 95% CI = 57.7–69.5%) and “Other mechanisms” (22.6% of weighted cases, 95% CI = 17.8–28.7%). The most common injuries were strains or sprains (33.2% of weighted cases, 95% CI = 27.8–39.5%) and fractures (28.1% of weighted cases, 95% CI = 24.3–32.4%). The most common body parts injured were the wrist (13.2% of weighted cases, 95% CI = 9.7–18.0%) and lower leg (12.9, 95% CI = 9.7–17.2%).

### Group T1: senior tennis-related cases

For senior tennis-related cases, the NEISS data contained n = 1363 unweighted records (weighted N = 74,932, 95% CI = 55,581–101,021). This group was 64.5% (95% CI =57.6–71.4%) male and the mean (weighted) age was 71.0 (95% CI = 70.6–71.5). The mean age is slightly inflated by cardiovascular/syncope cases (excluded from the tennis injury subset cases described below in group T2), the mean age for those cases was 72.4 years (95% CI =71.7–73.0).

### Groups P3 and T2: senior pickleball and tennis-related injury comparisons

Among players ≥60 years of age, the excluded non-injury cases accounted for 11.1% (3607/32,591) and 21.5% (16,096/74,932) of the pickleball and tennis-related cases, respectively. For seniors (≥60 years), NEISS contained a weighted total of 28,984 pickleball injuries (95% CI = 19,463–43,163) and 58,836 tennis injuries (95% CI = 44,861-77,164) from 2010 to 2019. Although tennis had more players in the 80+ age group, the mean age for both sports was similar, 69.5 years for pickleball (69.2 and 69.8 years for female and male players) and 70.7 years for tennis (69.4 and 71.6 years for female and male players).

Compared to tennis, pickleball had a higher prevalence of female players at 46.2% (95% CI = 41.6–50.9%) while tennis had 40.3% female players (95% CI = 36.3–44.4%). The univariate comparisons between the two sports for seniors ≥60 years are shown in Table 2.

**Table 2:** Univariate findings among senior pickleball and tennis injuries (weighted)

Characteristics	Senior Pickleball Weighted Count (95% CI)	Senior Pickleball Variable Proportion	Senior Tennis Weighted Count (95% CI)	Senior Tennis Variable Proportion
**Age Group (years)**				
60–69	15,738 (10,361-23,905)	54.3%	29,054 (21,987-38,392)	49.4%
70–79	11,804 (7841-17,771)	40.7%	22,391 (16,819-29,809)	38.1%
80+	1442 (749–2778)	5.0%	7391 (5434-10,053)	12.6%
**Sex**				
Male	15,588 (10,354-23,467)	53.8%	35,102 (26,953-45,716)	59.7%
Female	13,396 (8899-20,166)	46.2%	23,734 (17,402-32,369)	40.3%
**Treatment Year**				
2010	353 (241–517)	1.2%	5587 (2761-11,306)	9.5%
2011	289 (114–735)	1.0%	4434 (1844-10,663)	7.5%
2012	171 (33–872)	0.6%	6237 (3004-12,949)	10.6%
2013	596 (101–3521)	2.1%	4733 (2063-10,858)	8.0%
2014	916 (516–1628)	3.2%	5298 (2376-11,814)	9.0%
2015	3220 (1447-7166)	11.1%	7189 (2948-17,528)	12.2%
2016	4014 (1927-8360)	13.8%	6425 (2772-14,895)	10.9%
2017	4892 (2023-11,831)	16.9%	6473 (2824-14,836)	11.0%
2018	6301 (2725-14,566)	21.7%	5703 (2277-14,279)	9.7%
2019	8232 (2983-22,715)	28.4%	6757 (2554-17,875)	11.5%
**Treatment Month**				
Jan	3896 (2486-6105)	13.4%	5021 (3271-7706)	8.5%
Feb	4154 (2448-7051)	14.3%	8671 (5781-13,006)	14.7%
Mar	4669 (2643-8246)	16.1%	6069 (4045-9107)	10.3%
Apr	2853 (1677-4851)	9.8%	4545 (3236-6383)	7.7%
May	1632 (1027-2592)	5.6%	3605 (2624-4952)	6.1%
Jun	1291 (748–2230)	4.5%	4083 (3012-5535)	6.9%
Jul	990 (494–1986)	3.4%	4201 (3056-5776)	7.1%
Aug	1069 (604–1890)	3.7%	4165 (3181-5453)	7.1%
Sep	998 (572–1740)	3.4%	2926 (2165-3954)	5.0%
Oct	1830 (962–3483)	6.3%	4992 (3573-6975)	8.5%
Nov	2651 (1509-4657)	9.1%	5236 (3531-7764)	8.9%
Dec	2952 (2020-4313)	10.2%	5323 (3445-8224)	9.0%
**Disposition Group**				
Released	26,839 (18,069-39,865)	92.6%	52,163 (39,608-68,697)	88.7%
Hospitalized	2145 (1212-3797)	7.4%	6382 (4493-9065)	10.8%
Other	0 (NA-NA)	0.0%	291 (104–814)	0.5%
**Primary Mechanism**				
Slip/Trip/Fall/Dive	19,384 (12,516-30,020)	66.9%	34,742 (26,270-45,946)	59.0%
Other mechanism	6057 (3822-9601)	20.9%	12,855 (9310-17,750)	21.8%
Undetermined/unknown	1504 (983–2301)	5.2%	4870 (3611-6567)	8.3%
Hit object	957 (541–1694)	3.3%	893 (535–1490)	1.5%
Heat-related illness	750 (324–1735)	2.6%	2931 (1633-5259)	5.0%
Hit with racquet/paddle	168 (47–606)	0.6%	882 (513–1517)	1.5%
Hit player	94 (17–504)	0.3%	402 (169–954)	0.7%
Hit with ball	70 (14–345)	0.2%	1262 (758–2102)	2.1%
**Primary Diagnosis**				
Strain, sprain	8991 (5403-14,961)	31.0%	15,336 (11,075-21,238)	26.1%
Fracture	8797 (5816-13,307)	30.4%	13,834 (10,245-18,679)	23.5%
Contusions, abrasions	3205 (2029-5062)	11.1%	5748 (3959-8347)	9.8%
Internal injury	2599 (1413-4784)	9.0%	6465 (4816-8678)	11.0%
Other	2508 (1552-4052)	8.7%	8613 (6084-12,194)	14.6%
Laceration	1449 (884–2375)	5.0%	5084 (3624-7133)	8.6%
Dislocation	897 (500–1611)	3.1%	1495 (917–2436)	2.5%
Concussion	270 (87–843)	0.9%	835 (446–1562)	1.4%
Hematoma	268 (98–732)	0.9%	591 (15–403)	1.0%
Avulsion	0 (NA-NA)	0.0%	246 (85–713)	0.4%
Dental injury	0 (NA-NA)	0.0%	16 (2–114)	0.1%
Derma/conjunct	0 (NA-NA)	0.0%	76 (11–537)	0.1%
Hemorrhage	0 (NA-NA)	0.0%	78 (286–1220)	0.1%
Nerve damage	0 (NA-NA)	0.0%	388 (138–1092)	0.7%
Poisoning	0 (NA-NA)	0.0%	31 (8–123)	0.1%
**Body Region**				
Upper extremity	10,145 (6571-15,662)	35.0%	19,207 (14,649-25,184)	32.7%
Lower extremity	8005 (5149-12,445)	27.6%	13,102 (9488-18,093)	22.3%
Head/neck	5258 (3529-7833)	18.1%	13,727 (10,722-17,572)	23.3%
Trunk	4827 (3081-7561)	16.7%	9756 (7012-13,575)	16.6%
All of body	750 (324–1735)	2.6%	3028 (1700-5393)	5.1%
**Eye Injury**				
Non-eye injury	28,825 (19,333-42,979)	99.5%	58,000 (44,150-76,196)	98.6%
Eye injury	159 (48–522)	0.5%	835 (486–1435)	1.4%

**Fig. 3:**
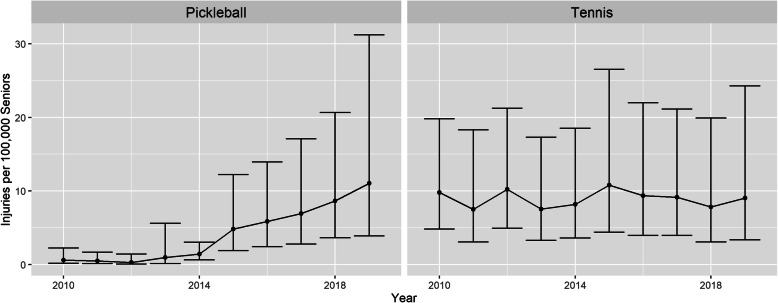
Senior injuries by sport and year per 100,000 of the U.S. senior population (weighted)

Figure 3 shows the rate of injuries per 100,000 seniors by year by sport. Each year includes error bars representing the 95% CI. The annual number of senior pickleball injuries grew steadily and significantly during the study period with 92.0% of cases occurring between 2015 and 2019. By contrast, tennis injuries stayed relatively constant. By 2018, the annual number of reported senior pickleball-related injuries reached parity with senior tennis-related injuries.

To identify the significance of this trend, the annual rate of senior pickleball injuries to tennis was tested. Adjusting for the sampling design using methods described by Thomas Yokota (Yokota n.d.) showed statistically significant evidence of a linear trend (*p*-value < .001). There is also weaker evidence for a quadratic trend (p-value = .096) and a cubic trend (p-value = .063). The “segmented” R package was used to identify potential breakpoints, estimated to be 2012 and 2015. The period between 2010 and 2012 had a beta of −0.51 and a *p*-value of 0.19, meaning there was no trend in senior pickleball compared to tennis injuries from 2010 to 2012. The period between 2012 and 2015 had a beta of 1.79 and a *p*-value of 0.0001, meaning there was a positive trend in senior pickleball injuries relative to tennis injuries. This period had the steepest growth by annual percentage change (APC), where senior pickleball cases almost doubled each year (APC = 93.1%) compared to senior tennis cases. Finally, the period between 2015 and 2019 had a beta of 0.83 and a *p*-value < .001, meaning there was a positive trend in senior pickleball injuries relative to senior tennis injuries during this period, the annual percentage change for this period was less steep but still statistically significant (APC = 10.4%).

**Fig. 4:**
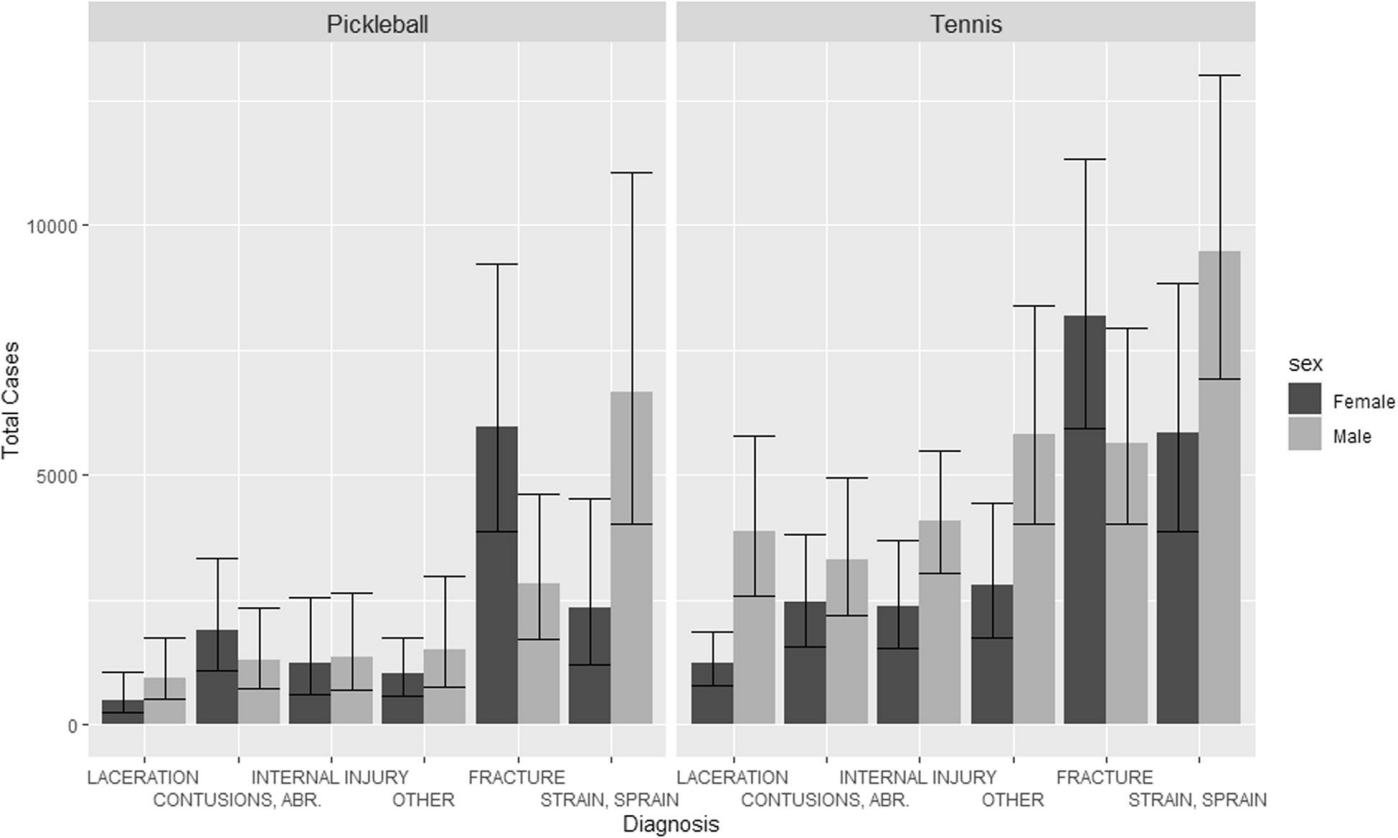
Top 6 most common diagnoses by sex, for senior pickleball and tennis injuries with 95% CI (weighted)

Among injury types, strains/sprains and fractures were the leading diagnoses for senior tennis and pickleball players and were highly gender imbalanced in both sports. Figure 4 shows the most common diagnoses by sex for seniors in both sports.

For senior women, Slip/Trip/Fall/Dive was the primary mechanism for 75.5% (95% CI = 72.2–82.8%) of pickleball injuries versus 64.4% (95% CI = 59.0–69.6%) of female senior tennis injuries. For senior men, Slip/Trip/Fall/Dive was the primary mechanism for 57.7% of pickleball injuries (95% CI = 47.3–68.1%) and 55.4% of tennis injuries. For both sports, male sprain/strains were mostly caused by an “other mechanism” which typically involved a movement-related injury. The most common body part injured from a male strain/sprain was the lower leg (which usually referred to either the calf or the Achilles tendon). For senior men, the lower leg accounted for 39.8% of pickleball strains or sprains (95% CI = 28.2–51.5%) but just 21.9% of tennis strains or sprains (95% CI = 15.2–28.5%).

Odds Ratios from survey-adjusted simple logistic regression were used to measure the associations between gender and diagnoses discussed above. In pickleball, women were over three-and-a-half times more likely to suffer a fracture (OR 3.7 95% CI = 2.3–5.7) compared to men and nine times more likely to suffer a wrist fracture (OR 9.3 95% CI 3.6–23.9). Both patterns were present but less extreme for senior tennis injuries, where women were just under three times more likely to suffer a fracture (OR 2.7 95% CI = 2.1–3.6) and five times more likely to suffer a wrist fracture (OR 5.0 95% CI = 2.9–8.7).

In pickleball senior women were over twice as likely to be injured from a Slip/Trip/Fall/Dive mechanism (OR 2.5 95% CI 1.5–4.3), while in tennis senior women were only slightly more likely to be injured from a Slip/Trip/Fall/Dive mechanism (OR 1.5 95% CI = 1.1–1.9). In both cases, the likelihood of a fracture among women was independent of whether a Slip/Trip/Fall/Dive was the primary mechanism.

Senior pickleball males were three and a half times more likely than females to suffer a strain or sprain (OR 3.5 95% CI 2.2–5.6), while no such difference was observed among senior tennis players (OR 1.1 95% CI 0.8–1.5). For both sports, the lower leg was by far the most common body part injured when males suffered a strain or sprain, but almost never for women. Male senior pickleball players were seven times more likely to injure their lower leg (OR 7.1 95% CI 2.9–22.0) compared to senior female pickleball players. Senior tennis injuries were similar but less extreme; men were twice as likely to injure their lower leg (OR 2.1 95% CI 1.1–3.8) compared to senior female tennis players.

Eye injuries were rare among seniors in both sports. Most eye injuries were caused by being hit with the ball. Eye injuries were more common among seniors playing tennis (1.4% of injuries in tennis vs. 0.5% of pickleball).

Both sports follow a similar seasonal pattern where injuries peak during the first 3 months of the year, then drop off until October.

## Discussion

Senior pickleball injuries presenting to U.S. emergency departments have increased dramatically in recent years as the sport’s popularity has exploded. By 2018 the national annual estimated number of senior pickleball injuries reached parity with the estimated annual number of senior tennis injuries. Both sports show pronounced seasonal distribution. We speculate that regional differences in the sport’s popularity in conjunction with seasonal and regional climate influences and seasonal relocation of many senior players (so called “snowbirds”) all play roles in this pattern.

Among senior pickleball cases, females were more likely to be diagnosed with a fracture than males, most commonly due to a Slip/Trip/Fall/Dive, and the wrist was the most common body part fractured. Plawecki and others have previously discussed the evidence of decreased bone density in older women as an overall fracture risk, (Plawecki et al. 2017; Daly et al. 2013) whereas the elevated female wrist fracture findings are notable. NEISS data are not specific enough to tell whether the paddle/racquet holding wrist was affected or not, so it is difficult to judge whether wrist protection is feasible and warranted as wrist protection on the dominant hand would likely interfere with comfort and play. This could be explored in future observational research.

Most senior injuries in both sports were caused by a Slip/Trip/Fall/Dive. Overall, the injury mechanism distribution among seniors for both sports are quite similar.

Eye injuries in both sports comprise a very small proportion of all reported ED injuries, but they can and do happen. This review extends the number of such case reports by several fold (Atkinson et al. 2020). Reviewing the narrative of each of the (n = 4 unweighted) cases showed that most pickleball eye injuries were caused by being hit with the ball and the narratives suggested that the pickleball eye injuries reported were generally less severe than the (n = 20 unweighted) tennis eye injuries. Eye injuries made up a higher proportion of all injuries among seniors playing tennis versus pickleball, 1.4% vs. 0.5% (weighted), respectively. If that difference were to hold in a larger sample, factors might include that the tennis ball is moving faster and is denser compared to a pickleball, carrying much more kinetic injury. An ongoing issue among pickleball players concerns the need for eye protection (Vitale and Liu 2020; Atkinson et al. 2020). Whatever the current use of eye protection is (unknown from these data), either using normal glasses or additional protective eyewear, the eye injury experience of senior pickleball players in these data do not exceed that of senior tennis players. However, eye injuries may not always present to ED’s, so without better surveillance, exposure, and measurement of protective gear used, a better understanding of eye injury risk is missing.

It was noted that many more cardiovascular related deaths were excluded from the tennis than the pickleball-related cases (13 vs 1, unweighted, respectively, while there were 2.5 times as many tennis injuries in the ten years of data). While these numbers are very small, and for reasons already discussed deaths were not a focus of this study, the larger proportion of tennis deaths does correlate with the larger percentage of non-fatal cardiovascular tennis cases removed prior to creating the senior injury subsets (18.6% versus 11.1% of the pickleball cases, unweighted). This suggests, very tenuously we acknowledge, that older tennis players may be more prone to cardiovascular events while playing than pickleball players. We speculate, should this pattern be confirmed, it might be due to the greater exertional demands of tennis on a larger court using a heavier racquet and ball with less doubles play than pickleball, but further research is needed. The difference in the sport specific proportion of non-fatal cardiovascular and syncope events was significant, however, suggesting that players with knowledge of any pre-existing cardiac disease who engage in both sports may preferentially, under clinical guidance, consider pickleball as less likely to aggravate cardiovascular risks.

This study had several advantages over the only other NEISS pickleball study: (Forrester 2020).

- Because there is no specific product or activity code for pickleball, a search of the NEISS narrative field is required to find cases. Searching only for the terms “pickle” and “ball” will miss misspellings with the text “pickel” (sic). We found several such cases.
- We accounted for mention in the narrative of multiple sports or activities and excluded them since the injury event could not be attributed solely to pickleball or the other activity.
- It is important from an injury prevention perspective to quantify how the injuries took place (the mechanism of injury) which we did from the narrative field.
- Previous work has recognized the popularity of pickleball injuries among seniors, but it is informative, especially for comparative purposes with other racquet sports, to look at the data from a senior-specific perspective since other sports may have very different age distributions. For example, among our NEISS pickleball-related cases, those 55 years and over made up 92.0% (95% CI = 89.9–94.2%) of the cases, whereas Chavinski (for the period 2010–2016) reported (Chevinsky et al. 2017) that only 39% of all NEISS tennis case occurred in patients aged 55 years and older and Gaw (for the period 1990–2011) reported (Gaw et al. 2014) only 23.8% of all NEISS tennis cases occurred in patients aged 56 years and older.
- As a new sport, cases have gone up markedly in recent years, so the addition of the most recently available two years significantly increased the size of the study sample.
- Previous pickleball and tennis NEISS studies did not exclude “non-injury” medical conditions such as syncope or cardiovascular events. This is not trivial in this active older cohort.

### Study limitations

The NEISS does not capture fatalities well and were very rare in this sample. Non-fatal cases treated outside the ED such as at urgent care centers, clinics, direct admits, school nursing and physicians’ offices, and those who did not seek medical treatment, are not captured by the NEISS. So, this data source underestimates medically attended and unattended injuries. Due to likely large geographic differences in the current uptake of these sports, the NEISS sample deign may not provide a precise accounting of actual injury rates. This may be especially true for pickleball, which is still new and expanding rapidly in different parts of the country.

NEISS case narratives are limited by the amount of detail included in the original medical records, abstractor consistency, and limited in character length. Because many different personnel enter information into NEISS, case narratives may not provide consistent information used to select cases and classify the primary mechanism of injury, even though the data collectors are trained. For a very small number of cases, we had to make reasonable judgements as to the intent of the abstractor. For example, a case where the person was noted as injured while playing “PICKLE” was excluded as a presumptive baseball-related activity, even though baseball was not coded. We also assumed if a tennis-related case was coded as “3284” and the narrative said the person had been hit by a tennis racquet that they were playing tennis if there was no place of injury mentioned. The NEISS does no case follow-up, so the amount of missed play and the long-term impacts are unknown.

Like most other reports using hospital-based injury surveillance system data, this study has no detailed and verified information on participation rates and quantified exposure (denominator data). No reliable data was available concerning pickleball participation by location, gender, or age. Nor are there any participation details available by type of play (indoors or outdoors, singles vs doubles, social vs competitive, etc.), time interval (weekly, monthly, or yearly), nor the total time (minutes) and intensity (e.g., steps, distance moved, speeds, strokes, calories expended, etc.) of play sessions important for accurate rate calculations and group comparisons. Lastly, frequently missing data for several fields in the NEISS data including race, Hispanic origin, and alcohol and drug involvement meant these variables could not be explored.

## Conclusions

NEISS is useful for describing the population-based epidemiology of different kinds of recreational injuries. However, careful case definitions are necessary to exclude false positives due to product and activity coding overlap and discern false negatives from both specifically (tennis) and broadly (pickleball) coded cases. They are especially important when examining records involving older populations since non-injury circulatory conditions related to activity/product codes of interest are often, and in this case, disproportionally included. The rapidly growing popularity of pickleball was strongly reflected in the reported injury trends. Senior pickleball injuries are increasing rapidly while senior tennis injuries have remained flat. The increase in pickleball injuries is almost entirely derived from the 60–79-year age group. Senior males were more likely than females to suffer a pickleball-related strain or sprain whereas women were more likely to suffer a fracture compared to men and much more likely to suffer a wrist fracture. Useful information for injury prevention and patient counseling was described, though true risk and exposure-based rate calculations requires observational research approaches outside the scope of this study.

## Data Availability

The raw data is available for query and download from the CPSC NEISS website: Highlights, Data and Query Builder (https://www.cpsc.gov/cgibin/NEISSQuery/home.aspx). Datasets used and/or analyzed during the current study are available from the corresponding author on reasonable request.

## Abbreviations

APC: Annual percentage change
CDC: Centers for Disease Control
CSV: comma separated values
CI: confidence interval
CPSC: Consumer Product Safety Commission
ED: emergency department
NEISS: National Electronic Injury Surveillance System
OR: Odds Ratio
U.S.: United States
